# Comparative Study of Nanosecond Electric Fields *In Vitro* and *In Vivo* on Hepatocellular Carcinoma Indicate Macrophage Infiltration Contribute to Tumor Ablation *In Vivo*

**DOI:** 10.1371/journal.pone.0086421

**Published:** 2014-01-27

**Authors:** Xinhua Chen, Shengyong Yin, Chen Hu, Xinmei Chen, Kai Jiang, Shuming Ye, Xiaowen Feng, Shifeng Fan, Haiyang Xie, Lin Zhou, Shusen Zheng

**Affiliations:** 1 Key Lab of Combined Multi-organ Transplantation, Ministry of Public Health, The Department of Hepatobiliary and Pancreatic Surgery. The First Affiliated Hospital, School of Medicine, Zhejiang University, Hangzhou, Zhejiang Province, China; 2 The Department of Pharmacy, Shandong Traditional Chinese Medicine University, Jinan, Shandong Province, China; 3 Colleges of Biomedical Engineering and Instrument Science, Zhejiang University, Hangzhou, Zhejiang Province, China; 4 Xinjiang Nanosecond Pulse Technology Institute, Urumqi, Xinjiang, China; Kanazawa University, Japan

## Abstract

**Background and Aim:**

Recurrence and metastasis are associated with poor prognosis in hepatocellular carcinoma even in the patients who have undergone radical resection. Therefore, effective treatment is urgently needed for improvement of patients' survival. Previously, we reported that nanosecond pulse electric fields (nsPEFs) can ablate melanoma by induction of apoptosis and inhibition of angiogenesis. This study aims to investigate the *in vivo* ablation strategy by comparing the dose effect of nanosecond electric fields *in vitro* and *in vivo* on hepatocellular carcinoma.

**Materials and Methods:**

Four hepatocellular carcinoma cell lines HepG2, SMMC7721, Hep1-6, and HCCLM3 were pulsed to test the anti-proliferation and anti-migration ability of 100 ns nsPEFs *in vitro*. The animal model of human subdermal xenograft HCCLM3 cells into BALB/c nude mouse was used to test the anti-tumor growth and macrophage infiltration *in vivo*.

**Results:**

*In vitro* assays showed anti-tumor effect of nsPEFs is dose-dependant. But the *in vivo* study showed the strategy of low dose and multiple treatments is superior to high dose single treatment. The macrophages infiltration significantly increased in the tumors which were treated by multiple low dose nsPEFs.

**Conclusion:**

The low dose multiple nsPEFs application is more efficient than high dose single treatment in inhibiting the tumor volume *in vivo*, which is quite different from the dose-effect relationship *in vitro*. Beside the electric field strength, the macrophage involvement must be considered to account for effect variability and toxicology *in vivo*.

## Introduction

Hepatocellular carcinoma (HCC) ranks the second leading cause of cancer death and causes over 600,000 new cases annually in China [1]. Liver transplantation or radical surgery is not always possible. Recently nanosecond pulsed electric fields (nsPEFs) have been proved effective in ablating different solid tumors [2]. Pulsed power is originally developed in World War II for weapons to store energy and release it very quickly to produce immediate high power. The applications have been extended to environment, agriculture, biology and medicine, its most important medical application of nsPEFs is tumor ablation. A number of studies have demonstrated that nsPEFs can stimulate apoptosis and ablate solid tumors such as melanomas [3], pancreatic cancer [4], basal carcinoma [5], hepatocellular carcinoma [6], cutaneous papilloma and squamous cell carcinoma [7].

The main characteristics of nsPEFs are their high power and low energy which lead to very little heat production. nsPEFs can produce highly compressed power, ultra short pulse, rapid rise time and high electric fields. The pulse is very short and penetrates into the cell before the plasma membrane is fully changed [8].

*In vitro* and *in vivo* experimental studies have broadened the understanding of the mechanisms by which nsPEFs mediate tumor eradication. nsPEFs can induce DNA damage, and eventually cell death through a variety of mechanisms including transient ion-permeable nanopores formation on both the plasma membrane [9] and intracellular membranes [10], apoptosis initiation and angiogenesis inhibition [11]. *In vivo* treatments try to kill as much tumor as possible while balancing damage to normal tissues around [12]. Many research groups have noticed that different cancer cells react differently to the same nsPEFs treatment, but the variability still remain incompletely understood. The critical experimental observation that the tumors *in vivo* are profoundly affected by the immunocompetence of the host remains generally ignored. Recently, in an allograft model system nsPEFs are found able to inhibit the growth of secondary tumors [13]. The relevance of this immune reaction induced by nsPEFs was noticed, which raise a new question: with the circulation and immune system, does the body show the same dose relationship of nsPEFs as in the *in vitro* assays? The effect of high and low dose nsPEFs are compared *in vitro* and *in vivo*. The involvement of macrophage *in vivo* is especially investigated.

## Materials and Methods

All studies on mice were conducted in accordance with the National Institute Guide for the Care and Use of Laboratory Animal. The animal protocol has been approved by the Committee of the Ethics of Animal Experiments of Zhejiang University.

### Cells and Animals

Human hepatocellular carcinoma cell line SMMC7721, HepG2, HCCLM3 and murine hepatocellular carcinoma cell line Hep1-6 were purchased from Chinese Academy of Science. Cells were grown in culture medium containing 10% FBS, penicillin (100 unit/mL), and streptomycin (0.1 mg/mL). These cell lines were incubated at 37°C in a humidified incubator under an atmosphere of 5% CO2.

BALB/c nude mice were purchased from Shanghai Experimental Animal Centre, Chinese Academy of Science. Experimental animals were kept in the central animal facility of the Zhejiang University School of Medicine and housed in laminar-flow cabinets under specific pathogen-free conditions with a 12 h light/dark cycle. All studies on mice were conducted in accordance with the National Institute Guide for the Care and Use of Laboratory Animal.

### Tumor Implantation and Tumor Volume Measurement

A metastatic human HCC animal model was established by implanting tumor tissue into the nude mouse subcutaneously. One injection was administered into each mouse. Mice were anesthetized by inhalation anesthesia. Before and after the treatment, tumors were measured and imaged by surface photography. Tumor volume was calculated by diameters measured with a caliper, using the formula V = 0.52×D1^2^×D2, where D1 and D2 are short and long tumor diameters, respectively [14].

### Pulse Generator and nsPEFs Parameters

A pulsor with Blumlein line configuration generated 100 nanosecond pulses. Pulses were applied at 0–60 kV/cm and 0.5 Hz. The pulse generator, voltage and pulsing pattern of nsPEFs were described previously [7]. The energy is written as

Where W is the energy (joule), τ is the pulse duration, V is the voltage across the electrodes (volt), R is the tissue resistance (Ω), and N is the pulse number. To study the dose effect *in vitro*, the pulse number was fixed to 10 and voltage ranged from 0–60 kV/cm [15].

### Electrode Design and Delivery

**Figure 1:**
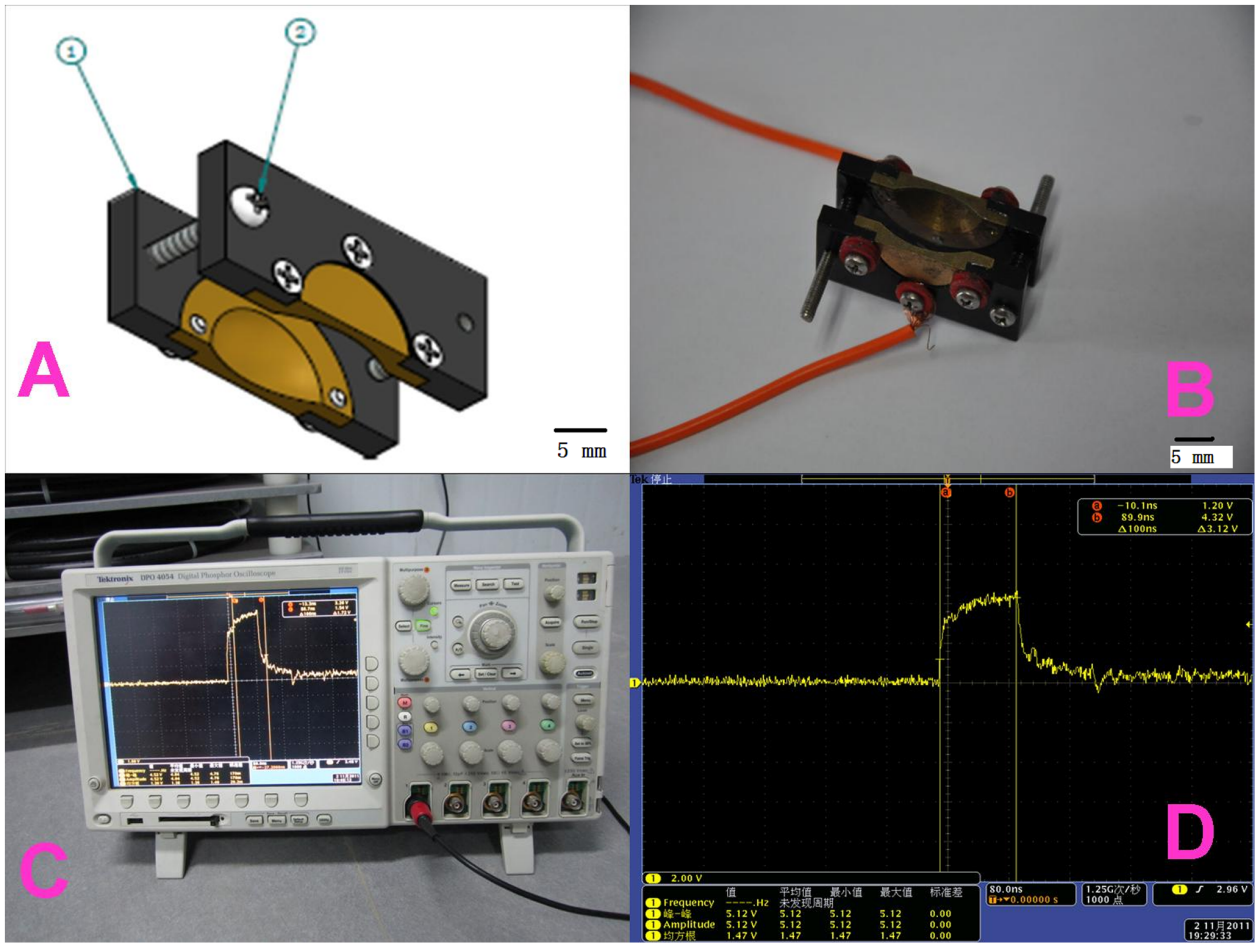
Electrode design and delivery. A is the design graph of the plate electrode. 1 is electrical insulating plate and 2 is adjustable screw. B is anterior view and C is lateral view of the real electrode. The delivery device consisted of two plates with a copper hollow hemi-sphere in the middle to hold the tumor (the inner diameter of 10 mm and a depth of 4 mm). The distance between two plates is adjustable by two screws to fit the tumor size. The plates made by electro insulating material can protect the normal skin and the copper sphere restricts nsPEFs application only to those tissues located inside the cavity. The field strength is nearly even to the center of hemisphere and drops in the edge.

The delivery device consists of two plates with a copper hollow hemi-sphere in the middle to hold the tumor (the inner diameter of 10 mm and a depth of 4 mm). The distance between two plates is adjustable by two screws to fit the tumor size. The space between the inner plate and tumor is filled with conductive gel to avoid spark in air. The plates made by electrical insulating material can protect the normal skin and the copper sphere restricts nsPEFs application only to those tissues located inside the cavity. The field strength is nearly even in the center of hemisphere and declines in the edge. The illustration of plate electrode is shown in Figure 1. (The design is shown in Figure 1A. The different profiles are shown in Figure 1B and Figure 1C).

### Experimental Group and nsPEFs Treatment

BALB/c nude mice were implanted HCCLM3 tumor blocks and then tumor diameter was measured. The mice were randomly divided into four groups to deliver the first treatment on the same day when tumor volume was around 0.4 cm^3^: control group (without nsPEFs treatment) (CT, n = 7), surgical resection groups (SR, n = 6), single treatment with 300 pulses group (S300, n = 7) and three treatments with 100 pulses group (T100, n = 8). T100 group had the second and the third treatment at one-day interval after intial treatment and on those days other groups only had anesthesia. Two weeks later the mice tumor volume in control group was beyond 2 cm^3^ and all mice were euthanized. Experimental animals in different groups were shown in figure S1.

### Cell Survival and Caspase Activation after Nanosecond Pulsed Electric Fields *in vitro*

Human hepatocellular carcinoma cell line SMMC7721, HepG2, HCCLM3 and murine hepatocellular carcinoma cell line Hep1-6 were cultured to log phase. Cells were harvested and re-suspended in fresh medium with 10% FBS, 1×10^6^ cells were placed in 0.2 cm gap cuvettes (Biosmith, Biorad) and exposed to 100 ns nsPEFs from 0, 10, 20, 30, 40, 50 and 60 kV/cm. After nsPEFs treatment, cells were seeded in 6-well plates (3.3×10^5^ cells/well). Twenty-four hours later, cells were detached and trypan blue negative cells were counted. The cell numbers were normalized as 100% to control cells. Or incubate for 60 minutes for caspase antibody fluorescent staining. The cells were counted and normalized to control cells as 100%. The experiment were repeated 3 times and shown as mean ± SD.

### Immunostaining of Tumor Sections *in vivo*

The dissected tumor samples for immunohistochemistry were fixed in phosphate-buffered neutral formalin, embedded in paraffin, and cut into 5-µm-thick sections. Tissue sections were deparaffinized, rehydrated and microwave-heated in sodium citrate buffer for antigen retrieval. Then, the sections were incubated with 3% hydrogen peroxide/PBS for 5 min, and blocked with SuperBlock solution (Pierce). Immunodetection was stained by fluorescent immunostaining of caspase (1∶500, Abcam, Hong Kong) and Mac 387(1∶200, Abcam, Hong Kong). All the slides were observed and photographed with an Axioskop microscope (Carl Zeiss, Oberkochen, Germany).

### Detection of Cell Metastasis and Motility Capability

At 1 h after nsPEFs treatment, the treated surviving cells at the same number were obtained to perform transwell assays based on transwell chambers (Millipore, USA). Briefly, the dead cells were removed and the rest cells were adjusted to the same number, suspended in serum-free medium and seeded into the upper chambers; 10%FBS was added to the bottom chambers. After 24 h, the cells on the upper side were removed with a cotton swab, while the cells on the bottom side of the filter were fixed, stained and counted. Wound assay reflecting tumor cell migration capability was done in 24-well plates with scratches introduced to the subconfluent cell monolayer by a plastic pipette tip. After 12, 24 and 48 hours, the scratch area was photographed and the wound distance between edges was measured and averaged from 5 points per 1 wound area. Experiments were repeated in triplicates.

### Statistical Analysis

Statistical analysis was performed with SPSS 15.0 for windows (SPSS, Chicago, IL, USA). Quantitative variables were expressed as means ± SD and analyzed by one-way ANOVA followed by Tukey's test. Results were considered statistically significant at *P*<0.05.

## Results

### 1. Cell Death and Caspase Activation Caused by nsPEFs are Dose Dependent *in vitro*

**Figure 2:**
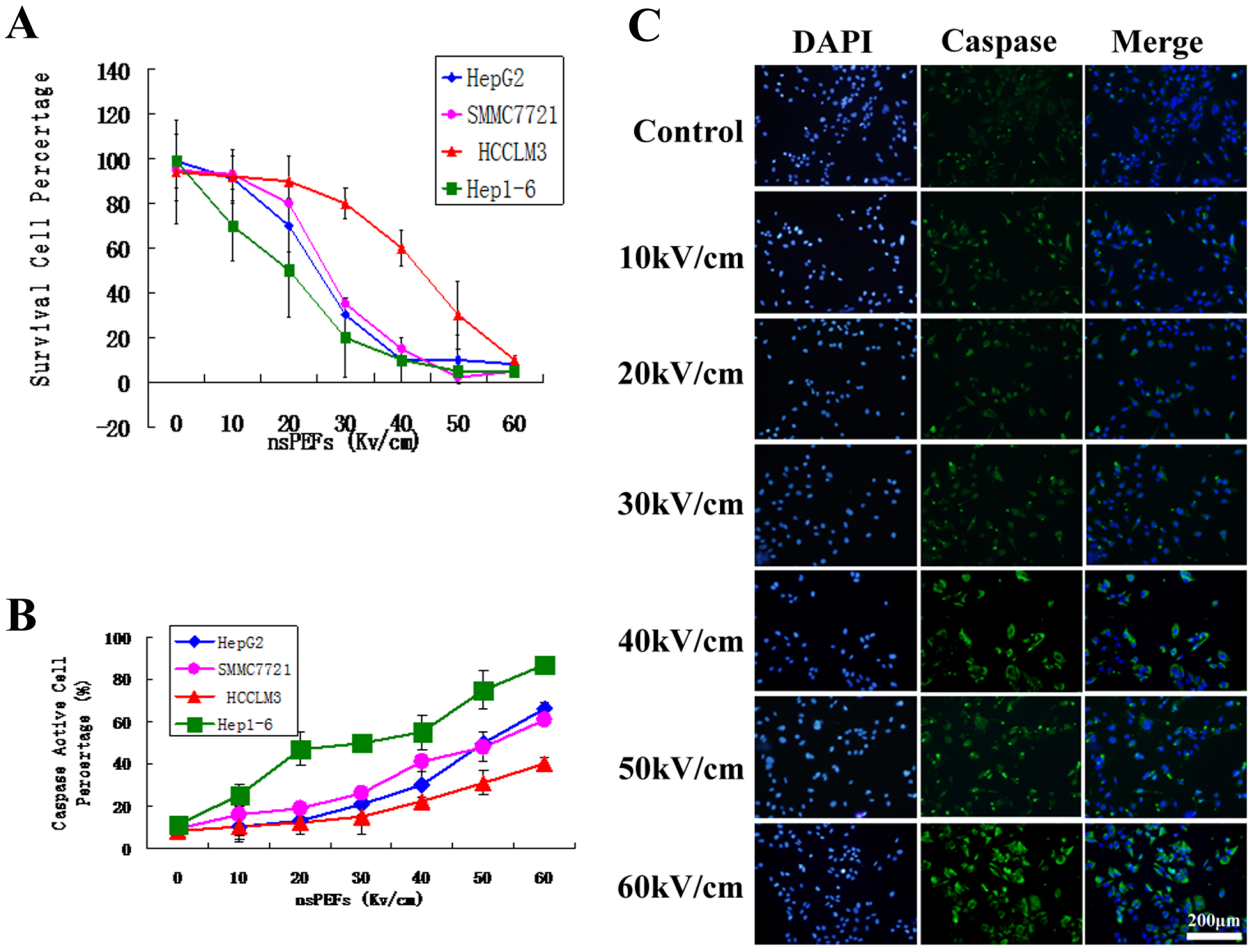
Cell death and caspase activation caused by nsPEFs are dose dependent *in vitro*. Cells were harvested with trypsin and re-suspended in fresh medium with 10% FBS and then placed in 0.2 cm gap cuvettes. They were exposed to ten 100 nsPEFs from 0 to 60 kV/cm. 24 h survival cell were counted and normalized to control cells as 100% (Figure 2A). The different cell lines of HCC showed different nsPEFs-vulnerability (hep1-6>HepG2>SMMC7721>HCCLM3), but all of their cell death rate increased when the nsPEFs raised from 0, 10, 20, 30, 40, 50 to 60 kV/cm. The caspase activation rate was also dependent on the nsPEFs strength (Figure 2C the caspase activation rate of HepG2). Similar results were also observed in SMMC7721, HCCLM3 and Hep1-6 cells (data not shown).

Cells were harvested with trypsin and resuspended in fresh medium with 10% FBS and then placed in 0.2 cm gap cuvettes. They were exposed to ten pulses of 100 nsPEFs from 0 to 60 kV/cm. After 24 hours, surviving cells were counted and normalized to control cells as 100% (Figure 2A). The different cell lines of HCC showed varying nsPEFs-vulnerability (hep1-6>HepG2>SMMC7721>HCCLM3), but all of their cell death rate increased when the nsPEFs raised from 0, 10, 20, 30, 40, 50 to 60 kV/cm. The caspase activation rate was also dependent on the nsPEFs strength. When the strength increased from 0, 10, 20, 30, 40, 50 to 60 kV/cm, the caspase activation rate increased accordingly (Figure 2B and 2C). When the pulse duration and pulse number are fixed, the strength is the dominant factor in the dose-effect relationship.

### 2. The Migration and Motility Capability Inhibition is Dose-dependent *in vitro*

**Figure 3:**
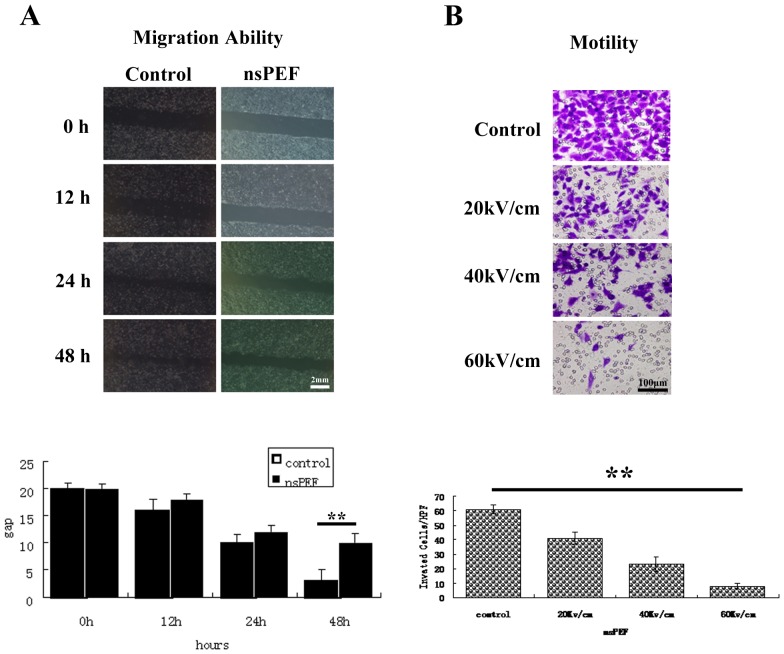
The motility and migration capability inhibition is dose-dependent *in vitro*. At 1×10^4^ cells/well were seeded in the the upper chamber in the serum-free media. The lower chamber were filled with media supplemented with serum. The chambers were incubated at 37°C with 5% CO2. The cells with migratory capability moved through the pores toward the serum below. After 24 hours, migrated cells moved to the lower well were stained with crystal blue and photographed under a phase-contrast microscope in five randomly chosen fields (100×magnification). The experiments were performed in triplicate wells and performed three times. The pulsed cells were also perform wound assay to reflect tumor cell migration capability with scratches. After 12, 24 and 48 hours, the scratch area was photographed and the wound distance between edges was measured and averaged from 5 points per 1 wound area. Experiments were repeated three times. The nsPEFs treated cells showed decreased migration capability in a dose dependant manner (Figure 3A HepG2 under 40 kV/cm). Quantification analysis indicates reduced motility capability in a dose-dependent manner (Figure 3B HepG2 under 40 kV/cm). Similar results were also observed in SMMC7721, HCCLM3 and Hep1-6 cells (data not shown).

Four samples of HCC cells were pretreated with nsPEFs as described above. The wound distance between edges was measured and averaged from 5 points per 1 wound area. The nsPEFs treated cells showed decreased migration capability in a dose dependant manner (*P*<0.05) (Figure 3A HepG2 under 40 kV/cm). Quantification analysis indicates reduced motility capability in a dose-dependent manner (Figure 3B HepG2 under 40 kV/cm). Similar effect of nsPEFs was also observed in SMMC7721, HCCLM3 and Hep1-6 cells (data not shown).

### 3. nsPEFs Ablate HCC Tumor *in vivo*

**Figure 4:**
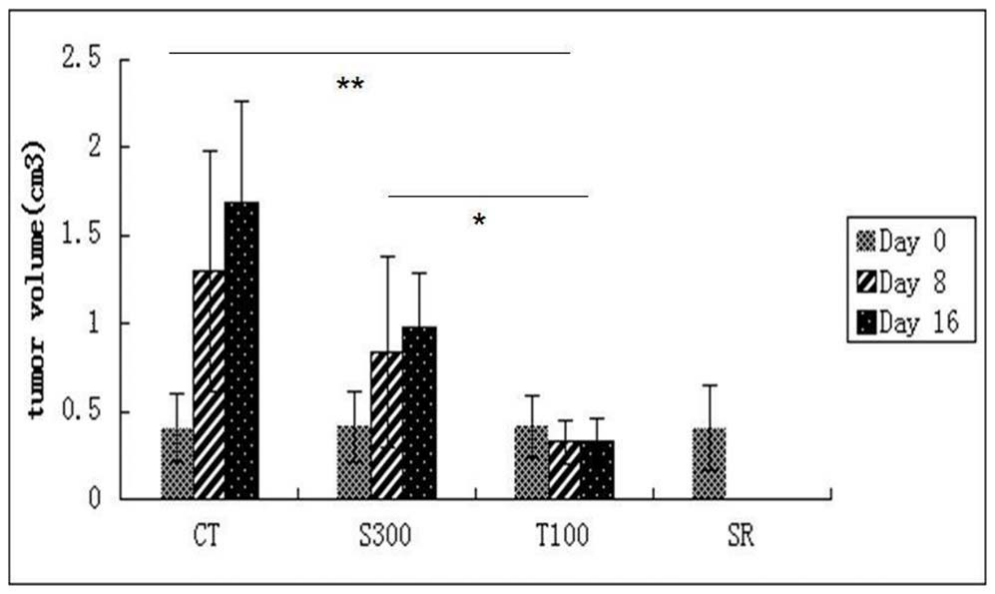
NsPEFs ablate HCC tumor *in vivo*. The mice were randomly divided into four groups. Control group (without nsPEFs treatment) (CT, n = 7), single treatment with 300 pulses group (S300, n = 7), three treatments with 100 pulses group (T100,n = 8); surgical resection group (SR,n = 6). The first nsPEFs treatment was deliver ed on the same day when the tumor volumes were around 0.4 cm^3^. The tumor volume on the nsPEFs treatment day, one week post treatment and the two weeks post treatment were calculated (Figure 4). Before the treatment (day 0), there was no significant difference in the tumor volumes among four groups (around 0.4 cm^3^.). On day 8 post nsPEFs treatment, tumor volume in T100 (0.42±0.11) cm^3^ was significantly decreased compared to CT (1.25±0.63) cm^3^ and S300(0.87±0.71) cm^3^. On day 16, tumor volume in T100 (0.40±0.14) cm^3^ was significantly smaller than that in CT (1.73±0.51) cm^3^ and S300(0.91±0.48) cm^3^.

The nsPEFs treatment began when tumor volume was 410±203 mm^3^ and terminated when tumor volume reached 2 cm^3^. In T100, S300 and SR group tumor volume was significantly inhibited (*P*<0.01) as shown in Figure 4. The tumors on the mice of the four gruops were shown in figure S1.

These results indicate that S300 and T100 had no lethal impact on tumor bearing mice. Both S300 and T100 can inhibit tumor growth, reducing tumor burden. Compared to S300, T100 showed a superior efficacy in reducing tumor volume.

### 4. Macrophage Infiltration Contributes to the Tumor Ablation *in vivo*

**Figure 5:**
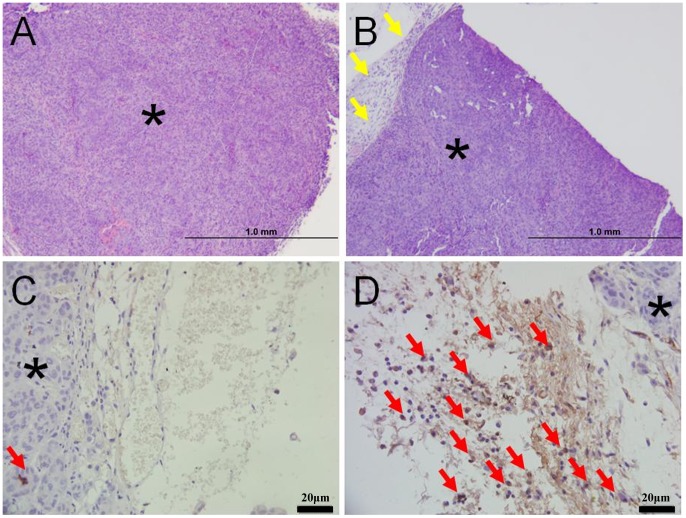
Macrophage infiltration contributes to the tumor ablation *in vivo*. The tumors were dissected with the attached tissue to study the macrophages infiltration. The star indicate the tumor cells. H&E stain showed the one day after nsPEFs treatment, tumor in T100 group shrank dramatically (figure 5B) compared with S300 (Figure 5A). Immunolabelling of macrophages in formalin-fixed, paraffin-embedded tissue, using the ABC method and haematoxylin counter stain (figure 5A and 5B). Tumor nodule in T100 group (figure 5B) was surrounded by a remarkable infiltration of inflammatory cells (The yellow arrow) compared with S300 group (figure 5A) which demonstrated few inflammatory cells. MAC387 antibody stained a moderate to high number of macrophages in the periphery of tumor from T100 group (figure 5D). Very few MAC387 positive cells (The red arrow) was seen in the edge of tumor from S300 group (figure 5C); The high density of MAC387 positive cells (The red arrow) found in the leading edge of tumor and especially within perivascular areas reflect host defensive macrophages recruited into the tumor vicinity. Beside the typical macrophage staining in figure 5, the macrophage infiltration in 28 mice were also detected. There was no macrophage infiltration in 7 mice of CT (0/7), mild macrophage infiltration in 4 mice of S300 (4/7), macrophage infiltration in T100 (intense in 6 mice, 6/8, and mild in 2 mice, 2/8).

H&E stain showed the one day after nsPEFs treatment, tumor in T100 group shrank dramatically (figure 5B) compared with S300 (Figure 5A). Tumor nodules in T100 group (figure 5B) was surrounded by a remarkable infiltration of inflammatory cells with significant amount of tumor cells compared with S300 group (figure 5A) which demonstrated few inflammatory cells. The MAC387-positive cells were found in the edge of tumor and especially within perivascular areas as shown in T100 group. There was no macrophage infiltration in 7 mice of CT (0/7), mild macrophage infiltration in 4 mice of S300 (4/7) and more macrophage infiltration in T100 (intense in 6 mice, 6/8, and mild in 2 mice, 2/8). The macrophage infiltration reflect host defensive reaction was activated in the tumor.

## Discussion

The curative treatment for HCC is surgical resection and liver transplantation, while most patients are in advanced stage and lose the chance of surgery. Other palliative treatments such as nsPEFs provide an opportunity [2].

nsPEFs deliver high energy to the targeted tumor without much heat production [16]. nsPEFs have good controllability by setting parameters, so the treatment is accurate with a well designed electrode. But in the tumor ablation experiment *in vivo*, the strategy has never been studied with strict controlled settings. The extremely high nsPEFs have been tried to ablate tumor in animal tumor model in which tumor volume is smaller than 2 cm^3^ [3], [6], [11]. But in clinical practice, early stage HCC patients with small tumors choose radical resection as the first line treatment. Those who come to localreginal ablation usually have tumors bigger than 3.5 cm in diameter. A single session of radiofrequency ablation (RFA) on HCC hardly attained complete ablation [2]. The big chunk of tumor requires rational strategy to obtain the complete ablation.

*In vitro* assays showed nsPEFs affect tumor cells in a dose-dependant manner. In the previous study [3], [4], thousands of pulses had been tried to kill tumor cells at once. But this comparative study *in vitro* and *in vivo* showed dose-effect relationship is not the same. Although the *in vitro* assays indicate HCC cell death, caspase activation and motility capability damage caused by nsPEFs are dose-dependant effect, *in vivo* study showed the different results. The single high dose treatment such as S300 does not cause the most significant anti-tumor effect. Low dose but multiple treatments such as T100 gain the significant tumor ablation.

Our study suggests that *in vivo* treatment strategy need to be further investigated. The host immune surveillance, the organ self-defensive barrier and electric conductivity of different tissues should all be considered in a whole system. The *in vitro* assays are not always accurate when used solely to predict the *in vivo* parameters and toxicity. For big tumors, nsPEFs ablation may reduce the tumor volume gradually instead of an immediate damage as radiofrenquency ablation does.

Human solid tumors are often infiltrated by lymphocytes, and the degree of the infiltration appears to be a favorable prognostic factor in hepatocellular carcinoma. This study indicates nsPEFs treatment cause a direct modulation of the tumor microenvironment through macrophage infiltration in the local tumors. The macrophages fight against local tumors and act as an effective immune response. This study proved nsPEFs treatment is a safe ablation method. With proper parameters and strategy, nsPEFs can ablate the hepatocellular carcinoma greatly with macrophage infiltration.

## Supporting Information

Figure S1 **The HCC tumor bearing nude mice were randomly divided into four groups.** Control group (CT) had no nsPEFs treatment, n = 7; single nsPEFs treatment group (S300) was treated single time with 300 pulses, n = 7, three treatments group (T100) were treated three times with 100 pulses, n = 8; surgical resection group (SR) had the radical resection of the tumor,n = 6.
(TIF)
