# Supplementary figures and images for: Comparative Study of Nanosecond Electric Fields In Vitro and In Vivo on Hepatocellular Carcinoma Indicate Macrophage Infiltration Contribute to Tumor Ablation In Vivo
Source: PLoS One. 2014 Jan 27;9(1):e86421. doi: 10.1371/journal.pone.0086421 (PMC3903538; doi:10.1371/journal.pone.0086421)

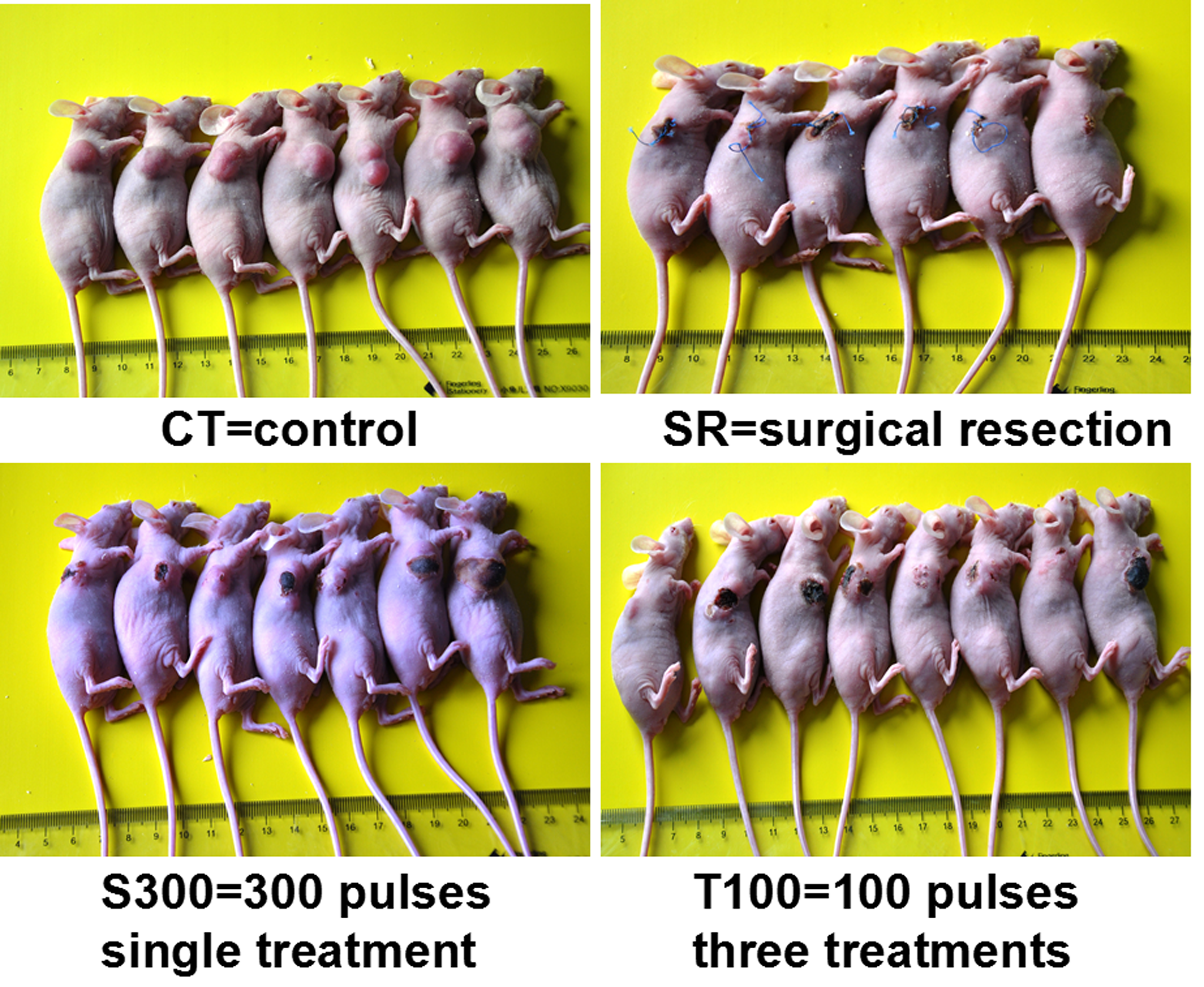

Supplement: Figure S1 — The HCC tumor bearing nude mice were randomly divided into four groups. Control group (CT) had no nsPEFs treatment, n = 7; single nsPEFs treatment group (S300) was treated single time with 300 pulses, n = 7, three treatments group (T100) were treated three times with 100 pulses, n = 8; surgical resection group (SR) had the radical resection of the tumor,n = 6. (TIF) [file pone.0086421.s001.tif]
